# Significant Enhancement of MgZnO Metal-Semiconductor-Metal Photodetectors via Coupling with Pt Nanoparticle Surface Plasmons

**DOI:** 10.1186/s11671-018-2573-7

**Published:** 2018-06-05

**Authors:** Zexuan Guo, Dayong Jiang, Nan Hu, Xiaojiang Yang, Wei Zhang, Yuhan Duan, Shang Gao, Qingcheng Liang, Tao Zheng, Jingwen Lv

**Affiliations:** 1 School of Materials Science and Engineering, Changchun University of Science and Technology, Changchun, 130022 China; 2 Research Center for Space Optical Engineering, Harbin Institute of Technology, Harbin, 150001 China

**Keywords:** MgZnO film, Ultraviolet photodetector, SPs, Electrode spacing

## Abstract

We proposed and demonstrated MgZnO metal-semiconductor-metal (MSM) ultraviolet photodetectors (UV) assisted with surface plasmons (SPs) prepared by the radio frequency magnetron sputtering deposition method. After the decoration of their surface with Pt nanoparticles (NPs), the responsivity of all the electrode spacing (3, 5, and 8 μm) photodetectors were enhanced dramatically; to our surprise, comparing with them the responsivity of larger spacing sample, more SPs were gathered which are smaller than others in turn. A physical mechanism focused on SPs and depletion width is given to explain the above results.

## Background

ZnO is an attractive wide direct band gap (~ 3.37 eV) oxide semiconductor featuring radiation hardness and environment friendliness. These characteristics make it suitable for the fabrication of short-wavelength optoelectronic devices, such as UV photodetectors. However, owing to the immaturity of p-type doping and other related solar-blind technology, the performance of ZnO-based UV photodetectors is still lower than expected. For the fabrication of high-performance ZnO-based UV photodetectors, a common and effective method is improving the material quality and optimizing the device technology, but this is usually a long-term process [1–7].

Recently, much attention has been paid to SPs for their fundamental scientific importance and promising practical applications. The SPs can be realized in coatings on the surface of metal NPs by magnetron sputtering. The metal NPs on the surface can enhance the scattering of the incident photons and make more photons reach the substrate, and thus, the absorption of the photons can be enhanced [8–18]. In many recent studies, Ag nanoparticles are considered to be a better material. But Ag could have been oxidized at the ZnO-Ag interface to form a layer of silver oxide (AgO) eventually [19]. As a kind of novel and stability properties metal in the world, the platinum (Pt) element has been an important candidate of the plasmonic material, whose SPs lies in the UV range. In addition, a metal-semiconductor-metal (MSM) structure has been preferentially chosen for the MgZnO photodetectors, with the advantages of a planar device structure, fast photo response, and simplicity in fabrication process. However, there has been rather limited systematic investigation of the combined effects of barrier height and depletion width, even though it could promote the progress of practical application and perfect fundamental physics. In this work, the MgZnO UV photodetectors with different active layers and electrode spacings have been designed and fabricated.

In this paper, we fabricated MgZnO MSM UV photodetectors assisted with SPs prepared by the radio frequency magnetron sputtering deposition method. Most importantly, the responsivity of the photodetectors were enhanced by sputtering metal Pt NPs on the surface of the device. In order to demonstrate SPs, then by comparing with electrode spacing of 3, 5, and 8 μm the responsivity of larger spacing, more SPs are smaller than others in turn. In theory, more SPs, more photo-generated electron-hole pairs are then created and the photo current is accordingly increased. To our surprise, due to the responsivity of larger spacing sample, more SPs were gathered which are smaller than others, demonstrating that this method is a powerful complement for the improving performance of photodetectors.

## Methods/Experimental

The MgZnO target was prepared by sintering mixture of 99.99% pure MgO and ZnO powders at 1000 °C for 10 h in air ambient then was placed on a zinc target. (The two targets have connected closely by the high-temperature conductive tap. The diameter of Zn target is 7 cm.) Clearly, the MgZnO beam flow will be enclosed by the Zn beam flow, reducing the losing of Zn atoms effectively [20]. The composition of the MgZnO film can be controlled easily, even at high substrate temperature.

**Fig. 1:**
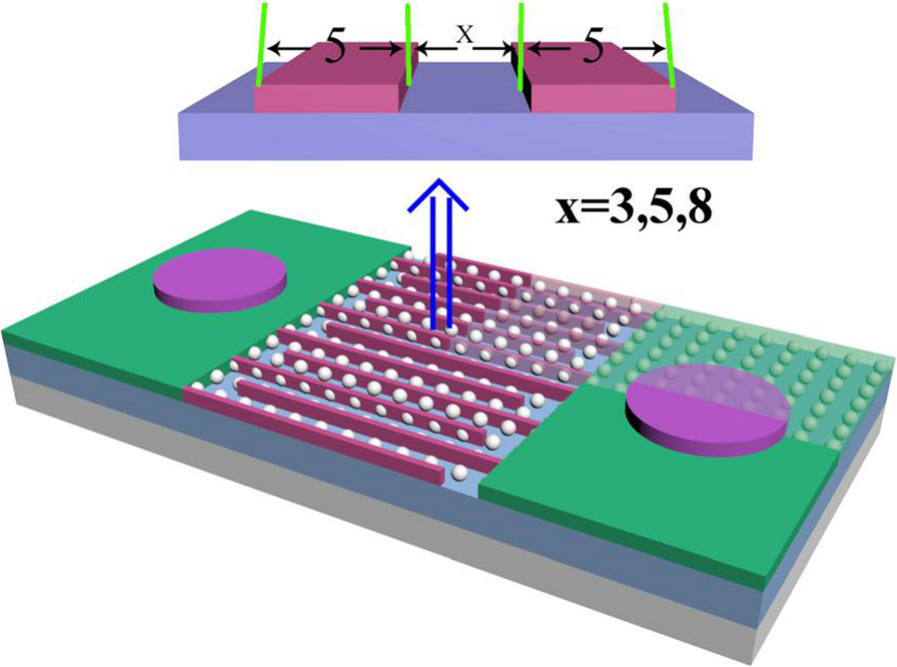
The 3D schematic of Mg_0.24_Zn_0.76_O UV PDs with MSM structure

The quartz substrates were successively cleaned 30 min with acetone, ethanol, and deionized water, then blown dry with air before deposition. The MgZnO film was grown on the quartz substrate first, with a total pressure of 3 Pa, a sputtering power of 120 W, at room temperature. Finally, the top Au finger electrodes were constructed through lithography and wet etching, which were 500 μm long and 5 μm wide with 3-, 5-, and 8-μm spacing, and the sum of finger pairs was 15 (Fig. 1 shows the schematic of photodetector).

The phase identification of the MgZnO film is characterized by the Rigaku Ultima VI X-ray diffractometer (XRD) with Cu Kα radiation (λ = 1.54184 Å) at 40 kV and 20 mA. A PerkinElmer Lambda 950 Spectrometer is used for the absorbance spectra in the wavelength range from 200 to 700 nm. The current-voltage (I-V) characteristics of the MgZnO photodetectors are measured under a 20 V bias using an Agilent 16442A Test Fixture. The spectral response for the MgZnO photodetectors is recorded using a Zolix DR800-CUST.

## Results and Discussion

**Fig. 2:**
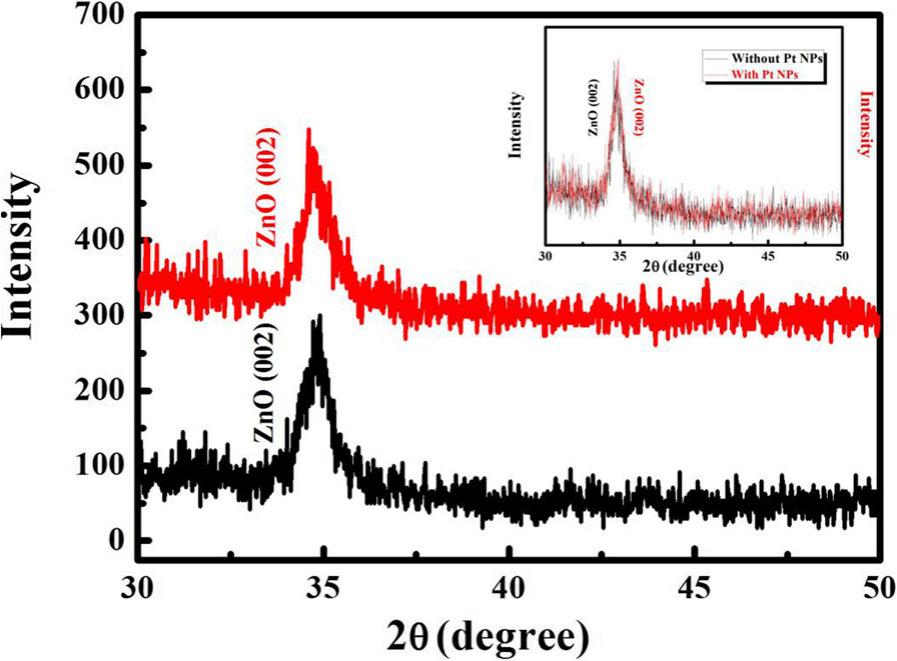
The XRD spectra of the Mg_0.24_Zn_0.76_O film

**Fig. 3:**
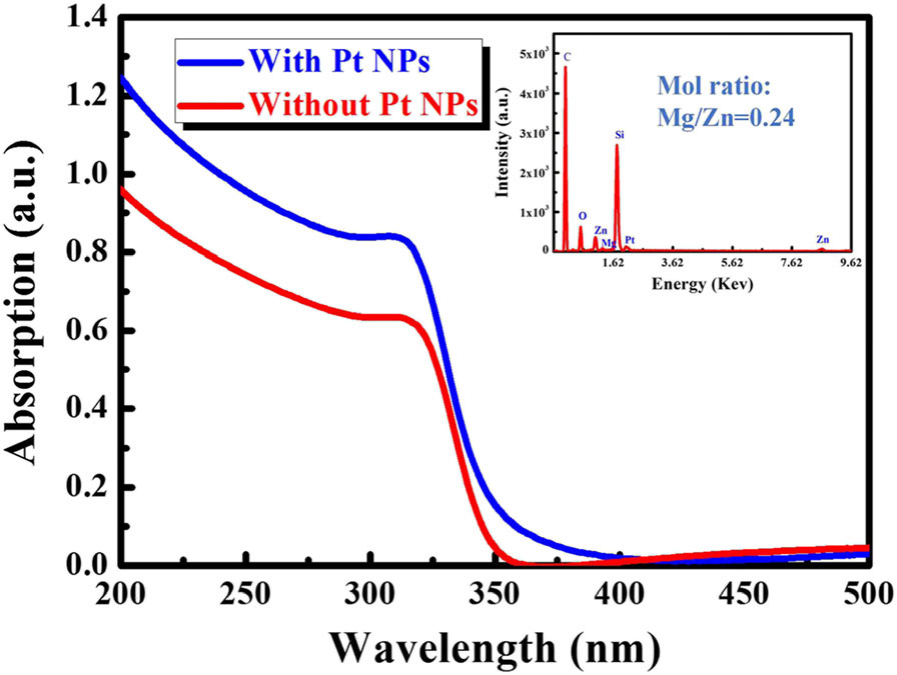
UV-visible absorption spectra of the Mg_0.24_Zn_0.76_O film

**Fig. 4:**
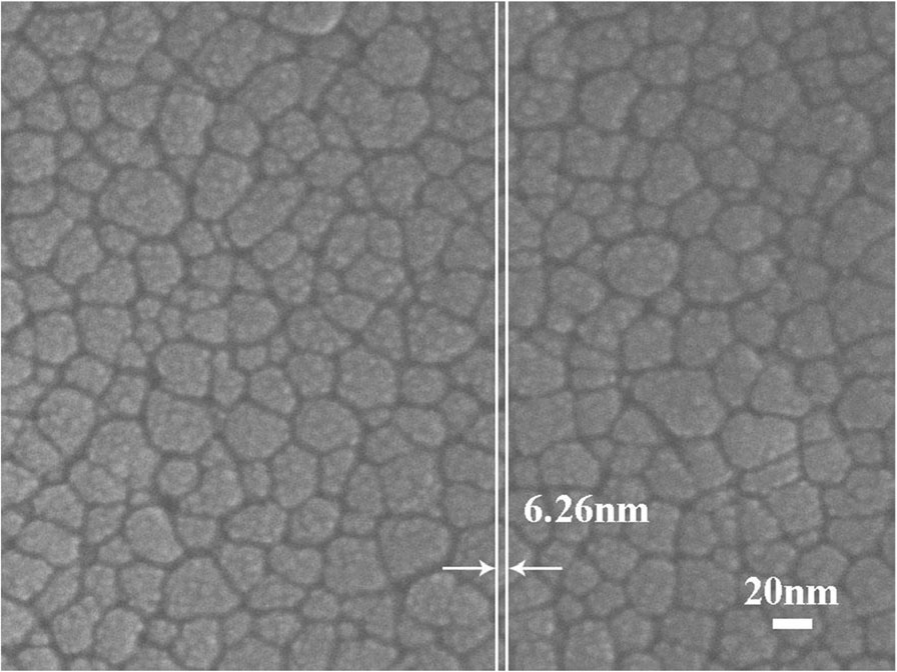
Plane-view SEM image of MgZnO surface with sputtering for 20 s, with Pt NPs

The XRD patterns of the MgZnO films in different sputtering time are shown in Fig. 2. Here is a diffraction peak located at about 34.84°, which can be indexed to the (002) plane of MgZnO, and it means that MgZnO films crystals are typically fabricated along the *c*-axis. The intensities of the without Pt NPs and with sputtering Pt NP MgZnO peaks are nearly the same, which can prove that the sputtering deposition Pt NPs deposited on the surface of MgZnO films and had no effect on the crystal quality of the films. Figure 3 illustrates the optical absorption spectra of the without Pt NPs and with sputtering Pt NP MgZnO films [21, 22]; the result suggests that the enhancement of absorption occurs for the detector with as-deposited Pt NPs due to SP modes. Compared with the pristine MgZnO film, the absorption of the MgZnO films coating with Pt NPs is enhanced in the spectrum range. Simultaneously, the MgZnO films were characterized by energy-dispersive spectrometer (EDS), and the magnesium concentration is about 24% (the inset of Fig. 3). Plane-view SEM image of MgZnO surface with sputtering for 20 s, with Pt NPs, are shown in Fig. 4. The average diameter of the Pt NPs is about 6.26 ± 0.50 nm.

**Fig. 5:**
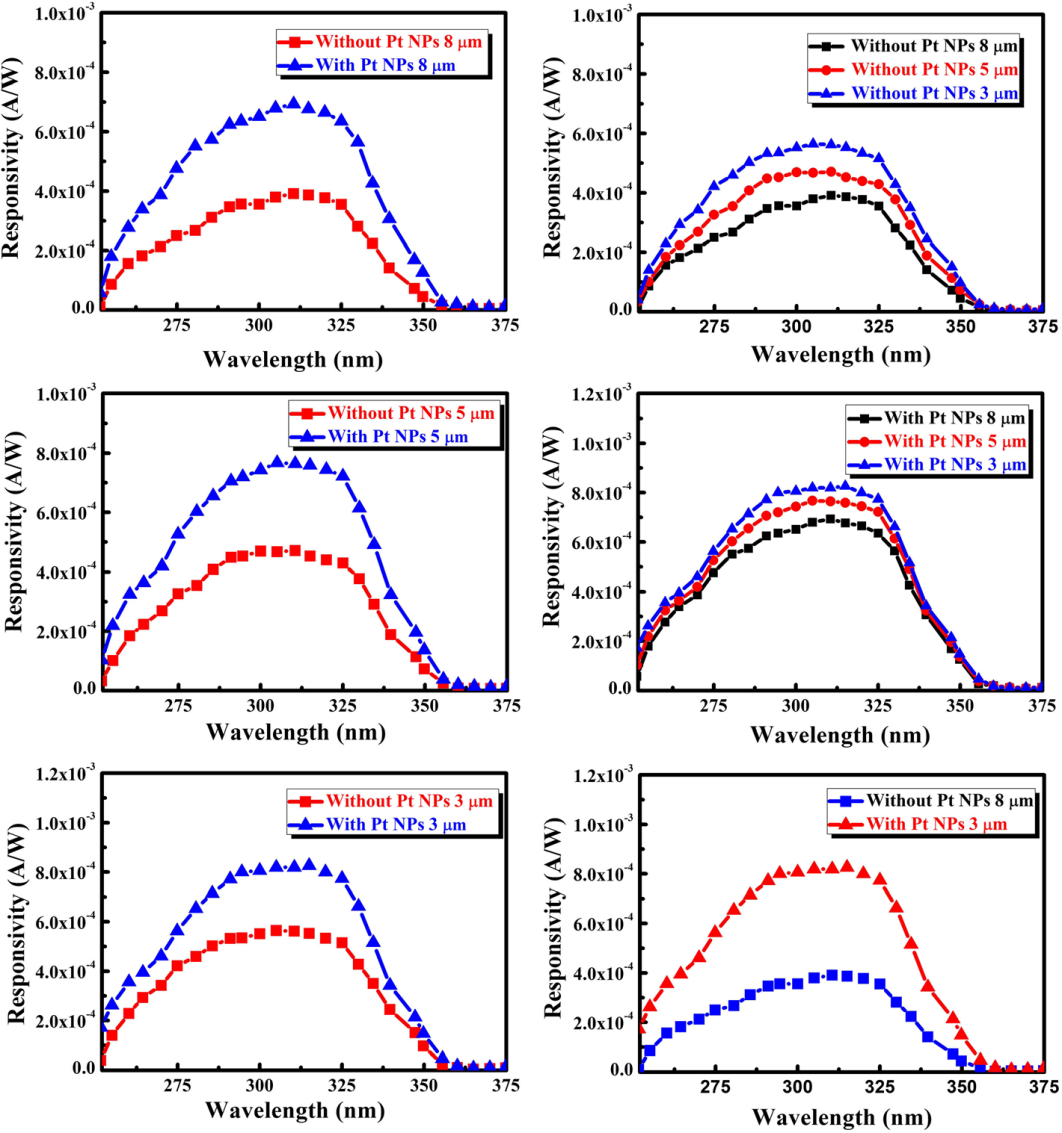
The responsivity of the MgZnO photodetectors (with different electrode spacing) versus incident light wavelength at 5 V bias

**Fig. 6:**
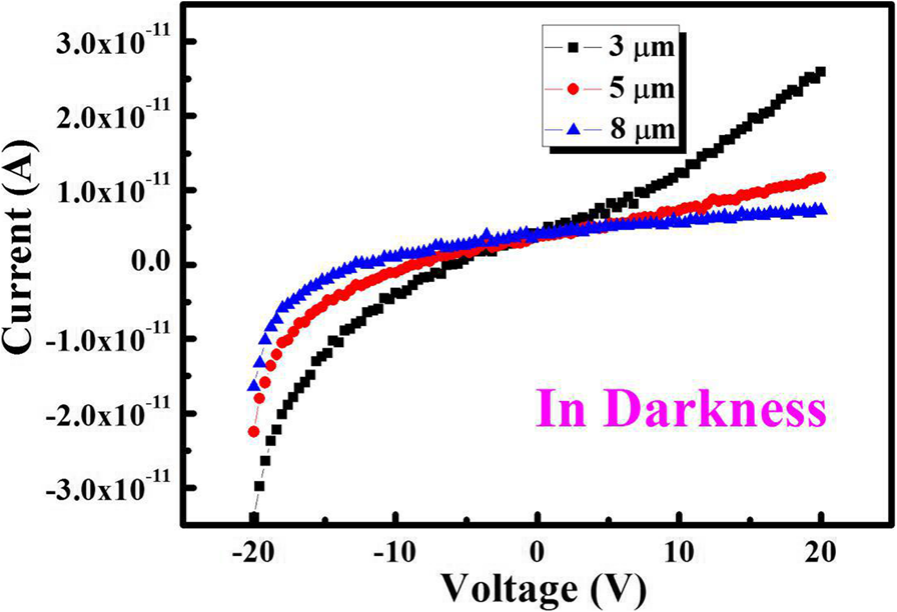
The non-linear I-V characteristics for the MgZnO photodetectors indicate that the classic Schottky metal-semiconductor contacts have been achieved

Figure 5 shows the responsivity of the MgZnO photodetectors (with different electrode spacing) versus incident light wavelength at 5 V bias. The responsivity enhancement tendencies were totally increased by decorating the Pt NPs. Notably, under the same conditions, all the photodetectors increase with decreasing electrode spacing (3, 5, and 8 μm). Therefore, the dominant component of the responsivity enhancement is the effect of the Pt NPs. The results indicate that the enhancement range of the responsivity can be controlled easily, which differs from conventional methods such as change bias voltage. To our surprise, due to the responsivity of larger spacing sample, more SPs were gathered which are smaller than others. In theory, because more SPs appear, more photo-generated electron-hole pairs are then created and the photo current is accordingly increased. The phenomenon is inconsistent with the theory. The non-linear I-V characteristics (shown in Fig. 6) for the MgZnO photodetectors indicate that the classic Schottky metal-semiconductor contacts have been achieved. It is also shown that the dark current is enlarged with the decreasing electrode spacing at the same bias, which can be explained by the depletion width of the metal-semiconductor junction.

**Fig. 7:**
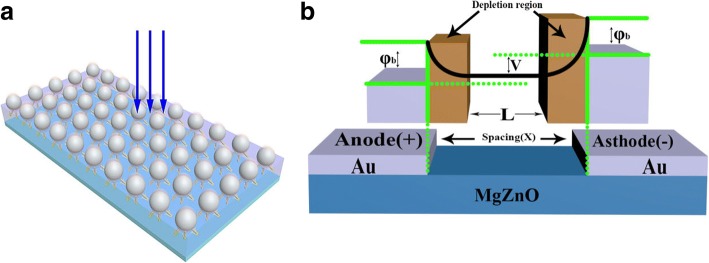
**a** The schematic of SPs. **b** The schematic of depletion width

To reveal the nature of the interesting phenomenon, two possible reasons are proposed as the cause which results between the enhanced responsivity and dark current: (1) In order to get the ideal combinatorial targets of MgZnO photodetectors, we use Pt NPs to modify the device again. The incident light of matching wavelength interacts with the metal NPs efficiently over scattering cross sections much larger than its geometrical cross-sections through coupling with SPs. The mechanism for the plasmonic scattering effect has been described in the literature. Thus, the scattered light then acquires a certain angular spread in the MgZnO layer. As a result, the incident light will pass several times through the semiconductor, increasing the effective optical path length. More importantly, increasing the optical path length can enhance light absorption. The photoresponse spectra of the with Pt NPs were gradually higher than that of without Pt NP devices (Fig. 7a shows the schematic of SPs). (2) The depletion width (*W*) explains why the responsivity of all MgZnO photodetectors increases with decreasing electrode spacing at the same bias. The depletion width can be described as [23]

1 $$ W={\left[2{\varepsilon}_0{\varepsilon}_1\left({\psi}_0+V\right)/{qN}_{\mathrm{d}}\right]}^{1/2} $$

where *ɛ*_0_ is the absolute dielectric constant, *ɛ*_1_ is the relative dielectric constant, *ψ*_0_ is the built-in potential, *V* is the bias voltage, *q* is the electron charge, and *N*_d_ is the donor concentration. As the electrode spacing increases, the area of the semiconductor thin film will increase, which refers to the effective resistance increases. *ɛ*_0_, *ɛ*_1_, *ψ*_0_, *V*, *q*, and *N*_d_ are invariants, thus resulting in widening as the electrode spacing increases, resulting in a decrease in the voltage acting on the depletion region. One can see only the bias effects of the depletion width; the voltage applied on the depletion region is that it reduces as the electrode spacing increases. Hence, any photo-generated carriers in this region would be swept out by the high electric field and drift to the metal electrodes. Thus, the amount of photo-generated carriers will increase, making the trend of the responsivity contrary to the spacing increase (Fig. 7b shows the schematic of depletion width). However, all the photodetectors increase with decreasing electrode spacing (3, 5, and 8 μm); under the same NP size and density, larger electrode spacing has more excited NPs; and then ability of near-field is coupled to the semiconductor is stronger. More photo-generated electron-hole pairs are then created, and the photo current is accordingly increased in theory. It is worth noting that the responsivity of all the photodetectors increase with decreasing electrode spacing (3, 5, and 8 μm) and bias voltage are constant. As mentioned above, the dominant factor focuses on depletion width to explain this interesting phenomenon. All the results reveal a practicable route to improve the responsivity of SPs. Here, as compared with other commonly used materials or previous photodetectors, lots of Zn atoms are losing during the growth process, which is due to the higher vapor pressure of Mg compared with Zn. It will have many defects formation into the films due to the deficiency of Zn atoms. The photo carriers will be compounded by the defects, and the responsivity of the solar-blind photodetectors will be reduced largely. In addition, owing to the losing of Zn atoms, the disorder and fluctuation of contents is difficult to avoid, and the tow tail phenomenon of the absorption edge will be following. As a result, the UV-visible rejection ratio will decay accompanying with the reducing of the detectivity. Consequently, the controlling of the stoichiometric ratio in the films may be a route to improve the performance of the MgZnO photodetectors. The SPs can be realized in coatings on the surface of metal NPs by magnetron sputtering. The metal NPs on the surface can enhance the scattering of the incident photons and make more photons reach the substrate, and thus, the absorption of the photons can be enhanced. In theory, more SPs, more photo-generated electron-hole pairs are then created and the photo current is accordingly increased. In order to demonstrate SPs by comparing with electrode spacing of 3, 5, and 8 μm the responsivity of larger spacing, more SPs are smaller than others in turn.

## Conclusions

In order to get the ideal MgZnO photodetectors, we fabricated MgZnO MSM ultraviolet photodetectors with different electrode spacings (3, 5, and 8 μm). Then, we have a novel approach (we use Pt NPs to modify the device) to increase the performance of the devices. To our surprise, by comparing with them the responsivity of larger spacing sample, more SPs were gathered which are smaller than others in turn. We detailed the wider depletion width, to explain the optimize responsivity, and we propose that the SPs of Pt NPs have enhanced the scattering of incident light, which is beneficial for further investigation in films photodetectors. Further study is underway to develop high-quality MgZnO UV photodetectors.

## Abbreviations

AgO: Silver oxide
EDS: Energy-dispersive spectrometer
MSM: Metal-semiconductor-metal
NPs: Nanoparticles
SPs: Surface plasmons
UV: Ultraviolet
